# Functional Modification of Cellulose Acetate Microfiltration Membranes by Supercritical Solvent Impregnation

**DOI:** 10.3390/molecules26020411

**Published:** 2021-01-14

**Authors:** Irena Zizovic, Marcin Tyrka, Konrad Matyja, Ivana Moric, Lidija Senerovic, Anna Trusek

**Affiliations:** 1 Faculty of Chemistry, Wroclaw University of Science and Technology, Wybrzeze Wyspianskiego 27, 50-370 Wroclaw, Poland; marcin.tyrka@pwr.edu.pl (M.T.); konrad.matyja@pwr.edu.pl (K.M.); anna.trusek@pwr.edu.pl (A.T.); 2 Institute of Molecular Genetics and Genetic Engineering, University of Belgrade, Vojvode Stepe 444a, 11010 Belgrade, Serbia; ivanamoric@imgge.bg.ac.rs (I.M.); seneroviclidija@imgge.bg.ac.rs (L.S.)

**Keywords:** supercritical solvent impregnation, antibiofilm properties, *S. aureus*, *P. aeruginosa*, microfiltration membranes, thymol, cellulose acetate

## Abstract

This study investigates the modification of commercial cellulose acetate microfiltration membranes by supercritical solvent impregnation with thymol to provide them with antibacterial properties. The impregnation process was conducted in a batch mode, and the effect of pressure and processing time on thymol loading was followed. The impact of the modification on the membrane’s microstructure was analyzed using scanning electron and ion-beam microscopy, and membranes’ functionality was tested in a cross-flow filtration system. The antibiofilm properties of the obtained materials were studied against *Staphyloccocus aureus* and *Pseudomonas aeruginosa*, while membranes’ blocking in contact with bacteria was examined for *S. aureus* and *Escherichia coli*. The results revealed a fast impregnation process with high thymol loadings achievable after just 0.5 h at 15 MPa and 20 MPa. The presence of 20% of thymol provided strong antibiofilm properties against the tested strains without affecting the membrane’s functionality. The study showed that these strong antibacterial properties could be implemented to the commercial membranes’ defined polymeric structure in a short and environmentally friendly process.

## 1. Introduction

Supercritical solvent impregnation (SSI) is an advanced technique for impregnating solid matrices with active substances soluble in the supercritical fluid [1,2,3]. In SSI, a supercritical solution of the active substance is brought into contact with the substrate in a batch or semi-continuous process. The absence of surface tension in the supercritical state allows for easy penetration of the fluid into the solid matrix and its impregnation [2]. The process is environmentally friendly, with no waste generation, and is energy-efficient [1,2]. Carbon dioxide is the most used supercritical solvent owing to the favourable critical properties (31.1 °C and 7.38 MPa), availability, non-toxicity, and non-flammability. One of the advantages of SSI over other impregnation and encapsulation techniques is the possibility of delivering active substances throughout the whole volume of finished polymeric forms, e.g., SSI of hip and knee endoprosthesis with α-tocopherol [1,4].

Increased bacterial resistance to antibiotics and the appearance of multi-drug resistant (MDR) bacteria is one of the biggest threats to global health, food security, and development today [5]. The hospital environment is particularly identified as the key node in the bacterial transmission network and is the source of opportunistic antibiotic-resistant pathogens [6]. A recent study [7] reported the first extensive genomic characterization of microbiomes, pathogens, and antibiotic resistance cassettes in a tertiary-care hospital from repeated sampling of 179 sites. It identified MDR strains as being widely distributed and stably colonizing across sites. Comparisons with clinical isolates indicated that such microorganisms could persist in hospitals for extended periods (>8 years) and infect patients [7].

The problem of increased bacterial resistance to antibiotics led research toward the design of novel antibacterial mats. As a modern and promising technique, the potential of SSI has been explored in the last ten years in the design of materials with antibacterial properties [2]. In a considerable number of reports, thymol was used as an active component because of its strong antibacterial properties against Gram-positive and Gram-negative bacteria, excellent solubility in supercritical carbon dioxide (scCO_2_), and GRAS (generally recognized as safe) status given by the FDA (Food and Drug Administration) [2]. Cellulose acetate (CA) is a polymer obtained by the acetylation of cellulose, the most abundant natural polymer. It is generally recognized as a biodegradable polymer [8] and can be produced with various properties for broad applications in consumers’ product design. CA is also a polymer easy to impregnate with thymol by SSI [9,10,11]. Because of the establishment of hydrogen bonding between the hydroxyl group of thymol and CA functional groups, thymol loadings from several to 72% by weight may be obtained [9]. This phenomenon offers the possibility of creating a wide range of CA-based materials, for different applications, with thymol as an active substance [9]. In our previous study [10], SSI was successfully applied to the design of CA surfaces (films) with strong antibacterial activity to prevent biofilm formation in *Pseudomonas aeruginosa* and *Staphylococcus aureus*, which are identified as pathogens of critical and high priority, respectively, for the development of novel therapeutic approaches [12].

CA microfiltration membranes have a broad range of applications, from wastewater treatment, membrane bioreactors, protein and enzyme filtration, biological fluid filtration, diagnostics cytology, and so on. Microfiltration membranes with antibacterial activity are desired in many applications, especially in preserving a sterile environment. The sterile environment is needed in protecting and venting medicinal devices and equipment in hospitals; it is highly desirable in post-operative units and inevitable in surgical rooms [13,14]. Microfiltration membranes are also used in gas-diffusers in surgery wound ventilation to provide a sterile flow of carbon dioxide or air [13,15], where *S. aureus* is identified as the common cause of infections in clean surgical wounds [15,16].

There are several reports in the literature on the production of CA membranes loaded or modified with active substances. Baldino et al. [17] applied the scCO_2_-assisted phase inversion method to produce highly porous CA-curcumin membranes. The dope solution was prepared by dissolving CA in acetone, with the subsequent curcumin addition in the quantity of 10–20%wt of CA used. Supercritical drying of the solution was performed at 35–55 °C and 15–25 MPa using a dynamic mode (with scCO_2_ flow rate). Andrade et al. [18] applied the phase-inversion method to produce CA membranes containing different amounts of β-cyclodextrin stabilized silver nanoparticles. The membranes reduced the number of adhered *Escherichia coli* cells by up to 93.8% compared with the control. Achoundong et al. [19] reported the modification of CA films aimed for gas separation via grafting of vinyltrimethoxysilane to hydroxyl groups of CA, with subsequent condensation of hydrolyzed methoxy groups on the silane to form a polymer network. Although the grafting was performed to enhance the membranes’ selectivity in gas separations, silanization, especially by aminosilanes, may also be used to add antibacterial properties to the material [20].

This study aimed to prove the feasibility of adding antibacterial properties to commercial CA membranes as polymeric forms with defined structures by the SSI with thymol. We hypothesized that it was possible to incorporate a sufficient quantity of thymol into the polymer phase of CA commercial membranes by SSI (avoiding thymol crystallization), which would provide strong antibacterial activity. We also assumed that this sufficient thymol loading could be attained without the destruction of membranes’ structure and functionality that might happen as a result of the unavoidable polymer swelling. SSI was performed in a batch mode, and the influence of pressure and processing time on the thymol loading was investigated. The modified membranes were tested in a cross-flow filtration system, and their functionality was compared to the functionality of initial untreated membranes. The impact of the SSI on the membranes’ microstructure was investigated by a dual-beam scanning electron and ion microscopy. Antibiofilm properties of the obtained material were tested against *S. aureus* and *P. aeruginosa*, while the membranes’ blockage due to the contact with bacteria was investigated for *S. aureus* and *E. coli*.

## 2. Results and Discussion

### 2.1. Supercritical Solvent Impregnation (SSI)

**Figure 1:**
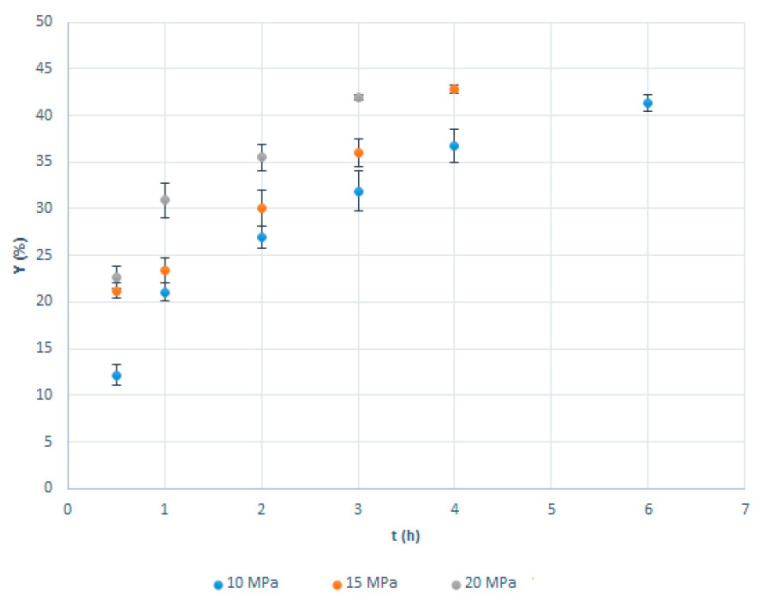
Impregnation yield as a function of the pressure and process time for supercritical solvent impregnation (SSI) of commercial cellulose acetate (CA) membranes at 40 °C.

The results of the SSI experiments are presented in Figure 1 and Table A1 (Appendix A). The experiments were performed in five replicates, with a standard deviation that can be considered low (Figure 1, Table A1). As can be seen, very fast impregnation was observed in the first hour of the process. Thymol loadings above 20% were obtained in 30 min of the SSI at pressures of 15 and 20 MPa. After one hour, impregnation yields of 21.1%, 23.4%, and 30.9% were reached at 10, 15, and 20 MPa, respectively (Figure 1). The maximal thymol loading was approximately 42% and was not dependent on the pressure. However, the time needed to achieve maximal loading was impacted by the pressure applied. The higher the pressure, the shorter the time required. At 10, 15, and 20 MPa, the maximal loading was obtained after 6, 4, and 3 h, respectively. It is worth noting that a slight wrinkling of the membranes with more than 30% of thymol was observed.

The faster impregnation at the higher pressure is the consequence of the thymol solubility in scCO_2_ increase with pressure [20] and interactions between the polymer and thymol. Namely, the pressure increase leads to the rise in scCO_2_ density and, consequently, enhances its solvating power. At 40 °C, thymol’s fraction in scCO_2_ increases from 0.0045 at 10 MPa to 0.0088 and 0.012 at 15 and 20 MPa, respectively [21], providing a larger driving force for the impregnation process at higher pressure. In some cases, as for thymol and polycaprolactone [22], when there is no strong interaction between the active molecule and polymer, higher solubility of the active substance in scCO_2_ might reduce its loading in the solid substrate (polymer). However, in this case, there is a large affinity of CA towards thymol owing to the establishment of hydrogen bonding between their hydroxyl groups. Therefore, SSI of CA with thymol is enhanced by the pressure increase, as previously reported in the literature [9,10].

**Figure A1:**
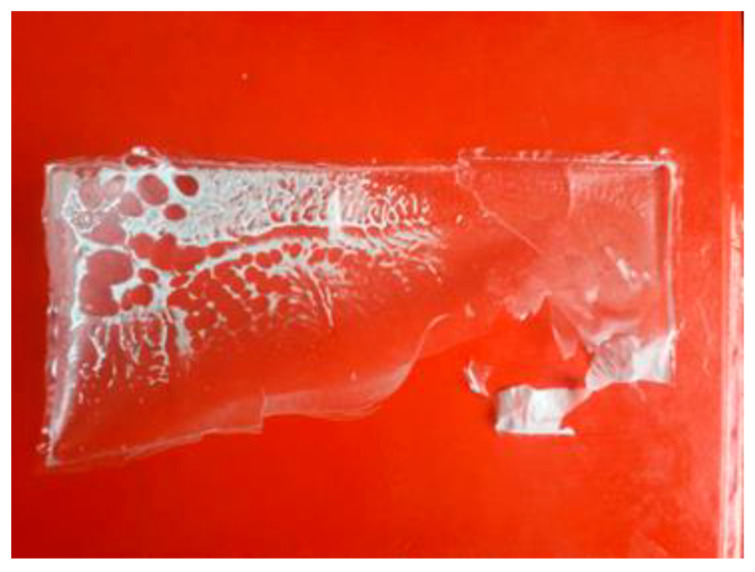
CA-based film with 10% of thymol obtained by the solvent casting method; unpublished data from the study Zizovic et al. [10].

Milovanovic et al. [9] investigated SSI of CA beads with thymol at 35 °C and pressures of 10 and 20 MPa. After two hours of impregnation, thymol loadings of around 4.5% and 27% were obtained at 10 and 20 MPa, respectively. This is a considerably slower impregnation rate in comparison with that observed in our study. The difference is most likely due to a substantially larger porosity of the commercial microfiltration membranes than CA beads. On the other hand, maximal beads’ loadings, regardless of applied pressure, were approximately 72%, when the beads’ shape was strongly affected [9]. In this study, impregnation yields of CA membranes higher than 42%, which might have been obtained through the extension of the impregnation time, were not explored because of the apparent membrane wrinkling. Zizovic et al. [10] examined the SSI of CA films prepared by the solvent casting method with thymol. Depending on the dope solution composition in the film preparation, thymol loadings in the range from 4.1% to 62.3% were achievable without significant disruption of the film’s appearance. It is important to stress that such high loadings of thymol are possible to obtain only by applying supercritical carbon dioxide. Conventional membrane and polymeric film preparation methods, such as the solvent casting method, can achieve several percent active substance loadings. Rodríguez et al. [23] prepared CA-based composite films with up to 2% of thymol by the solvent casting method. With larger thymol quantities, thymol crystallization occurs, as presented in Figure A1 (Appendix A). However, in SSI, thymol dissolved in scCO_2_ easily penetrates the polymer matrix because of the absence of surface tension in the supercritical phase and stays in it, distributed on the molecular level dowing to the establishment of hydrogen bonding.

### 2.2. Fourier-Transform Infrared (FTIR) Analyses

**Figure 2:**
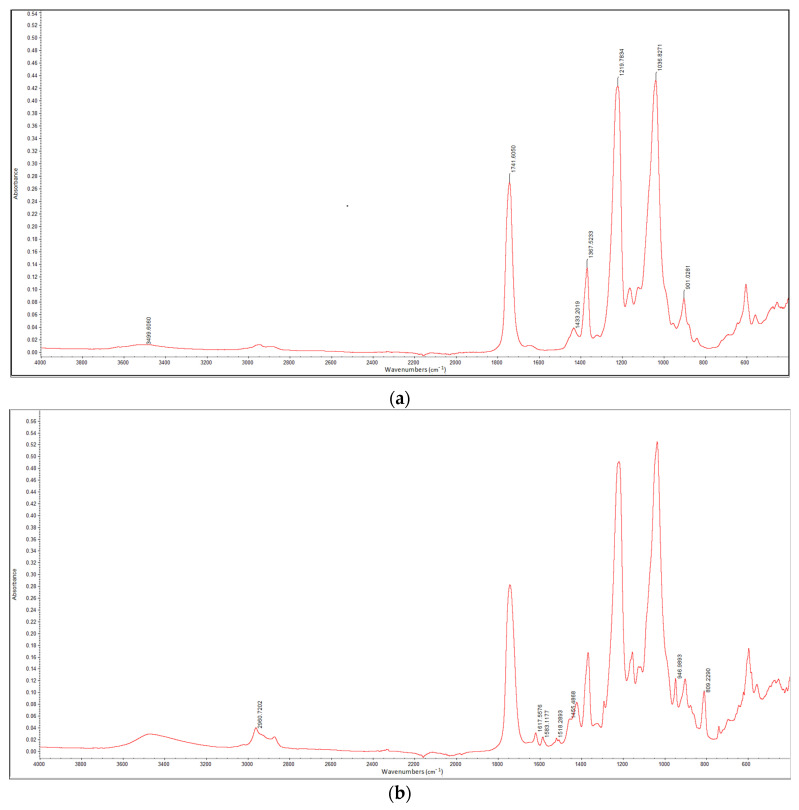
Fourier-transform infrared (FTIR) spectra of the neat CA membrane (**a**) and CA membrane with 20% of thymol (**b**).

To confirm the presence of thymol on the surface of impregnated membranes, FTIR analysis was performed. Spectra of the neat CA membrane and CA membrane with 20% of thymol are presented in Figure 2a,b, respectively. Characteristic peaks for cellulose acetate are observed in the spectrum of the neat CA membrane (Figure 2a). The broad absorption band at 3500 cm^−1^ is assigned to O-H stretching of the hydroxyl group [11,24,25,26,27,28]. The peak at 1741 cm^−1^ is ascribed to the stretching mode of the C=O group [11,25,26,27,28,29]. The bands at 1433, 1367, and 1219 cm^−1^ are assigned to the C-H bending, rocking, and wagging vibrations, respectively [11,18,20]. The peak at 1036 cm^−1^ originates from C-O-C (ether linkage) from the glycosidic unit, while the peak at 901 cm^−1^ is a characteristic of saccharide [11,24,26,27].

In the FTIR spectrum of the impregnated CA, new peaks appeared, indicating the presence of thymol. New peaks in the range 1455–1620 cm^−1^ are assigned to the phenol ring of thymol [11,30,31]. The peak detected at 809 cm^−1^ is attributed to out of plane aromatic C-H wagging vibrations [11,31], while a new peak observed at 946 cm^−1^ originates from out of plane aromatic C-H bending vibrations [32]. The peak at 2961 cm^−1^ is assigned to the C-H stretching of the thymol methyl group [31,33].

### 2.3. Structural Analyses by Scanning Electron Microscopy (SEM)

**Figure 3:**
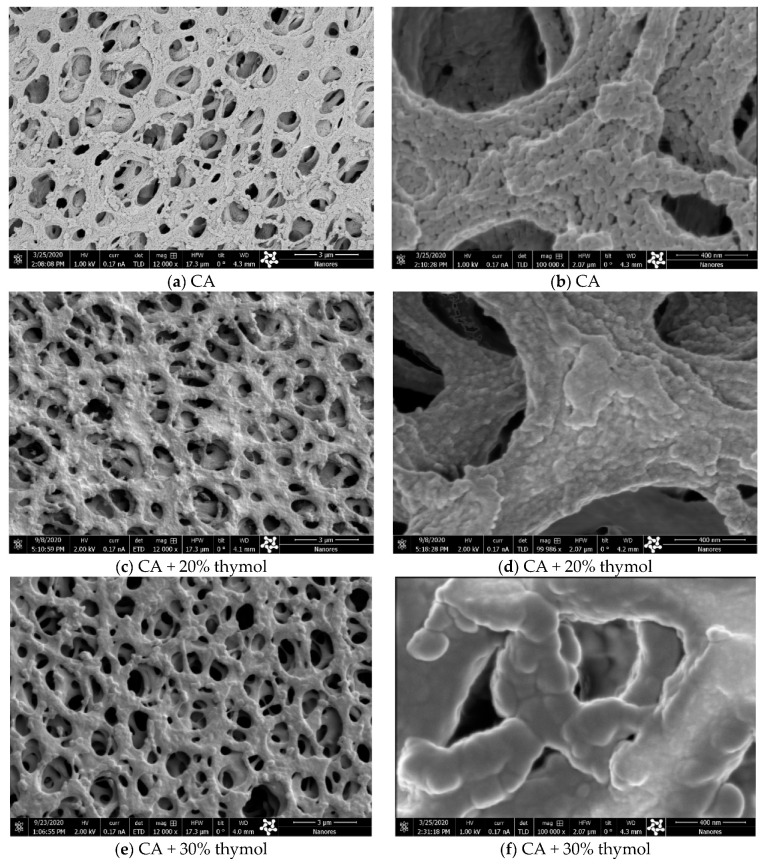
Scanning electron microscopy (SEM) images of membrane surfaces of the neat CA (**a**,**b**), CA with 20% of thymol (**c**,**d**), and CA with 30% of thymol (**e**,**f**) (bar = 3 µm for images **a**, **c**, and **e**; bar = 400 nm for images **b**, **d**, and **f**).

SEM analyses were employed to evaluate the possible effects of SSI with thymol on membranes’ structure. Namely, during SSI, newly established hydrogen bonds between CA and thymol weaken the existing electrostatic interactions between polymer chains and enhance the chains’ mobility [9,10,11]. Such unavoidable swelling might affect the membranes’ microstructure. SEM images of membrane surfaces for the neat CA membrane and membranes with 20% and 30% of thymol are presented in Figure 3. As can be seen in images 3*a*–3*d* (Figure 3), slight swelling of polymer occurred in the sample with 20% of thymol. Comparison of the micrographs 3*b* and 3*d* revealed that a bump-like neat polymer appearance (3*b*) was less pronounced in the impregnated sample (3*d*). However, a considerable polymer swelling was observed in the sample with 30% of thymol (micrographs 3*e* and 3*f*).

**Figure 4:**
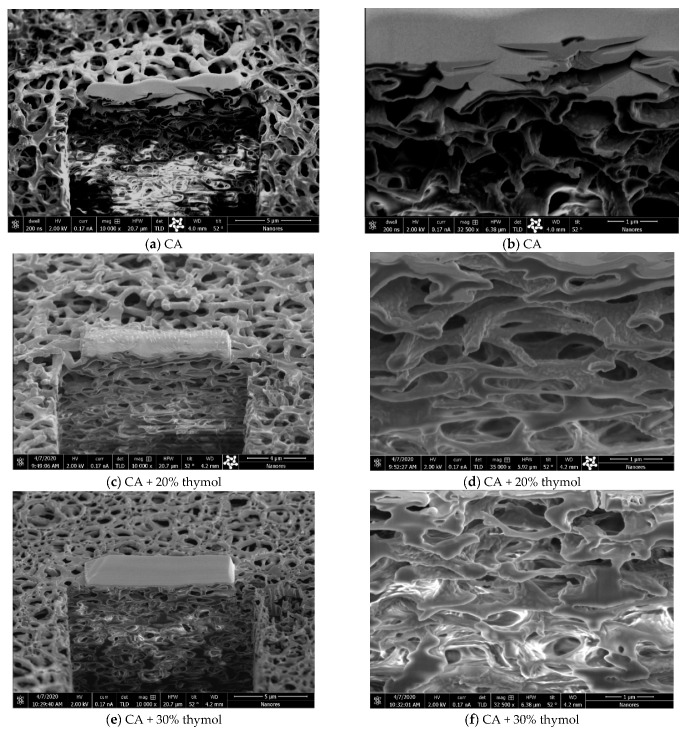
SEM images of membrane cross-sections of the neat CA (**a**,**b**), CA with 20% of thymol (**c**,**d**), and CA with 30% of thymol (**e**,**f**) (bar = 5 µm for images **a** and **e**; bar = 4 µm for image **c**; bar = 1 µm for images **b**, **d**, and **f**).

The possible effect of polymer swelling on the membrane microstructure was also studied by applying the energy-focused beam of gallium ions, which allowed for the sample cross-section during the SEM investigation. The cross-sections of the neat CA membrane and membranes with 20% and 30% of thymol are presented in Figure 4. Comparison of the micrographs 4*a*–*4d* showed that swelling occurred in the sample with 20% of thymol, but indicated that the membrane’s microstructure had been preserved. On the contrary, in the case of the sample with 30% of thymol, polymer swelling affected the membrane’s microstructure (micrographs 4*e* and 4*f*). This finding is in accordance with the observed slight wrinkling of membranes with more than 30% of thymol.

### 2.4. Membrane Anti-Biofilm Properties

**Figure 5:**
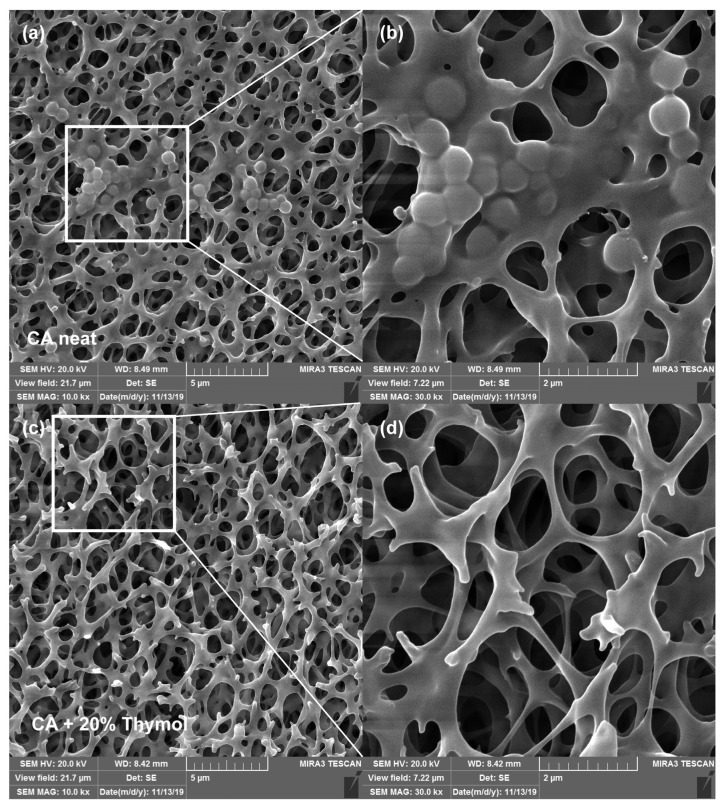
SEM micrographs of membranes’ surfaces incubated for 24 h with S. aureus ATCC 25923. (**a**) Neat CA membrane covered with bacteria. (**b**) Magnification of the squared region on the neat membrane containing bacterial biofilms. (**c**,**d**) CA membrane impregnated with 20% thymol without bacterial cells’ presence observed.

The anti-adhesion activity of thymol-impregnated CA membranes was first addressed against *S. aureus* biofilms. The thymol-impregnated and neat CA membranes were incubated with *S. aureus* ATCC 25923 and methicillin-resistant *S. aureus* ATCC 43300 (MRSA), and the formation of biofilms on their surfaces was assessed after 24 h. The SEM analysis of CA membranes’ surfaces revealed that the neat membrane was covered with *S. aureus* biofilms (Figure 5a,b), while bacterial attachment was completely inhibited by impregnation of a membrane with thymol (Figure 5c,d). Although *S. aureus* ATCC 43300 (MRSA) adhered to the neat membranes 10 times more than *S. aureus* ATCC 25923 cells, none of the bacteria have been recovered from the surface of both thymol-impregnated membranes (Table 1).

Thymol-impregnated CA membranes also exhibited anti-adhesive activity against *P. aeruginosa* compared with neat membranes (Table 1). The presence of 20% thymol reduced ten-fold *P. aeruginosa* PAO1 biofilm formation compared with neat membranes, while 30% thymol impregnation reduced bacterial adhesion 10,000-fold. The effect was less pronounced when compared with the thymol effect on *S. aureus* biofilm formation, but it was still statistically significant for both thymol concentrations.

This prominent anti-adhesion property of impregnated membranes is most likely achieved because of the strong antibacterial activity of thymol [34] by killing either bacteria that adhered to the membrane surface or those in close proximity upon thymol release [35]. According to the overall results, CA membranes impregnated with 20% of thymol were selected for further study as they showed strong antibiofilm activity against both tested species, while their microstructure, according to SEM images, remained preserved.

### 2.5. Membrane Testing in the Cross-Flow Filtration System

**Figure 6:**
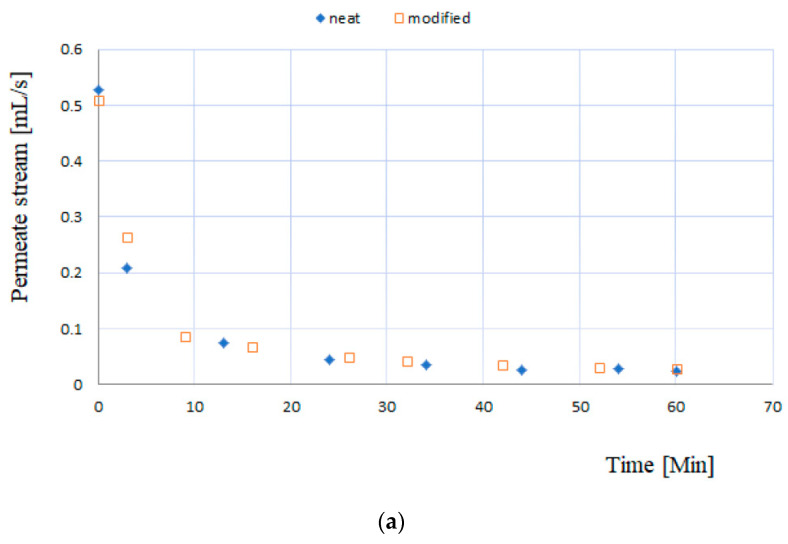

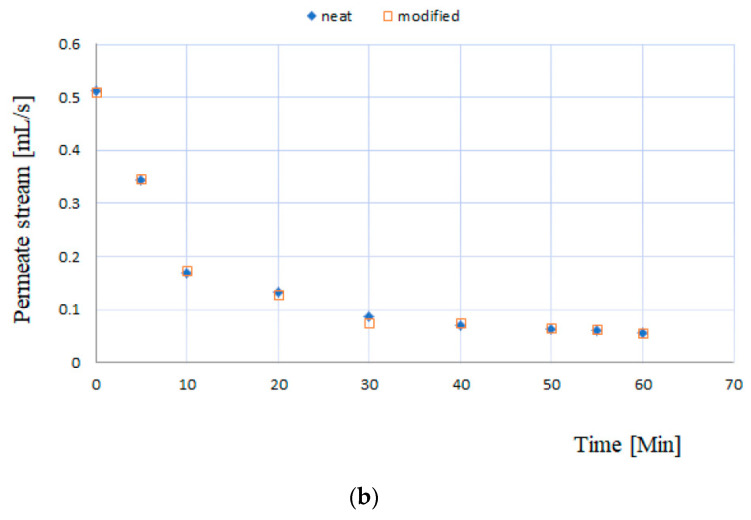
The change in permeate stream during *E. coli* culture broth filtration at 20 °C; mean values based on three measurements, maximal average relative deviation (RD) = 5.1%. (**a**) Initial cell concentration in feed 115.9 × 10^6^ CFU/mL, feed volume 300 mL, and ΔP = 0.2 MPa; (**b**) initial cell concentration in feed 25.2 × 10^6^ CFU/mL, feed volume 450 mL, and ΔP = 0.2 MPa.

The functionalities of neat and modified membranes (20% thymol) were tested in a cross-flow filtration system with *E. coli* culture broth. The filtration experiments were performed with two initial biomass concentrations, and the decline of the permeate stream was measured (Figure 6). As shown in Figure 6, the membranes behaved in the same manner, revealing no adverse effects of the SSI processing on the membrane’s functionality. The results also showed that thymol’s presence did not slow down the biomass deposition on the membranes’ surface during the cross filtration.

**Figure 7:**
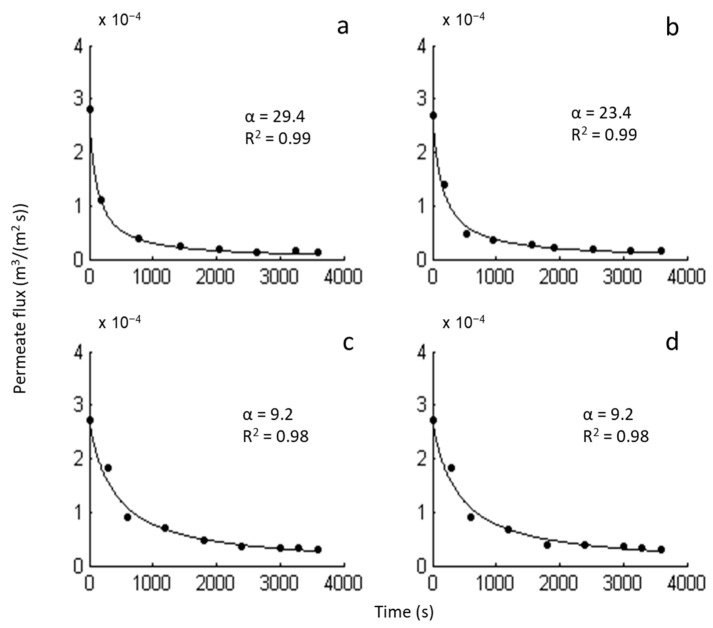
The permeate flux decline for the fixed value *β* = 1. (**a**) Neat CA membrane, cell concentration 115.9 × 10^6^ CFU/mL; (**b**) modified CA membrane, cell concentration 115.9 × 10^6^ CFU/mL; (**c**) neat CA membrane, cell concentration 25.2 × 10^6^ CFU/mL; (**d**) modified CA membrane, cell concentration 25.2 × 10^6^ CFU/mL.

**Figure A2:**
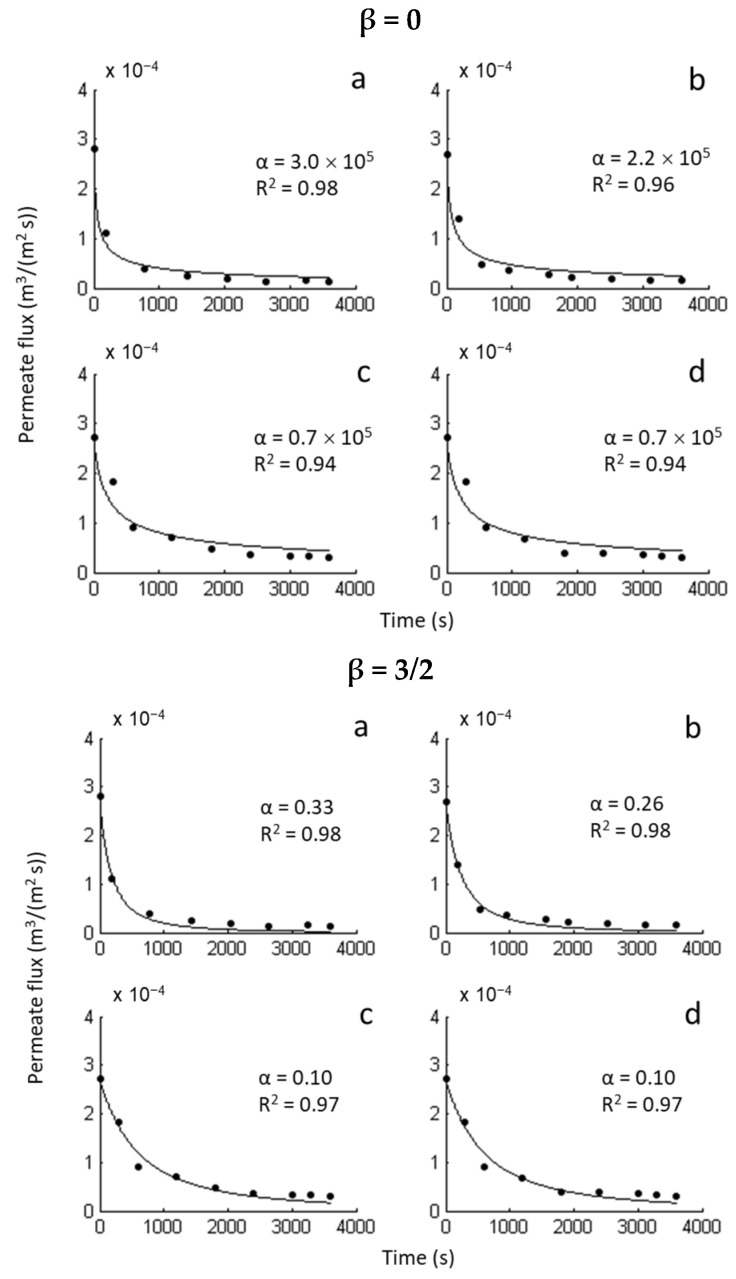

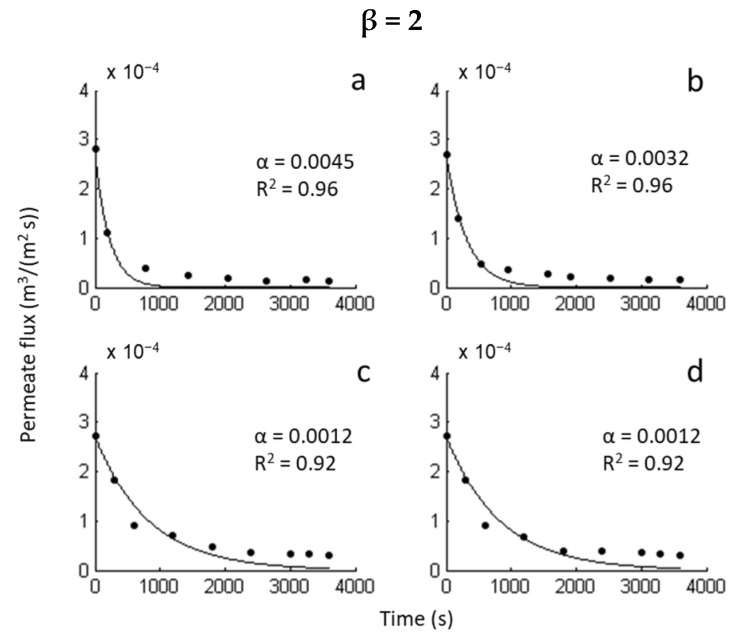
The permeate flux decline for the fixed value β. (**a**) Neat CA membrane, cell concentration 115.9 × 10^6^ CFU/mL; (**b**) modified CA membrane, cell concentration 115.9 × 10^6^ CFU/mL; (**c**) neat CA membrane, cell concentration 25.2 × 10^6^ CFU/mL; (**d**) modified CA membrane, cell concentration 25.2 × 10^6^ CFU/mL.

Mathematical modeling revealed that the second-order kinetics (*β* = 1) describe the filtration process most accurately (Figure 7). The modeling results for the other β parameter values (0, 3/2, and 2) are presented in Appendix B (Figure A2). The value of 1 for the parameter *β* implies the intermediate blocking mechanism [36,37]. The intermediate blocking phenomenon presumes that each particle can settle on other particles that previously arrived and have already been blocking some pores, or it can also directly block some of the membrane area [37]. According to this blocking mechanism, parameter *α* corresponds to the ratio of the blocked membrane surface and total porous surface of the membrane [36]. Considering the *α* values obtained by the modeling (Figure 7), it can be concluded that the presence of thymol contributed to the reduction of the blocked surface area in the case of a higher cell concentration (smaller *α* value for the modified membrane). In the case of a lower cell concentration, the modified and neat membranes behaved in the same manner (the same *α* values).

**Figure 8:**
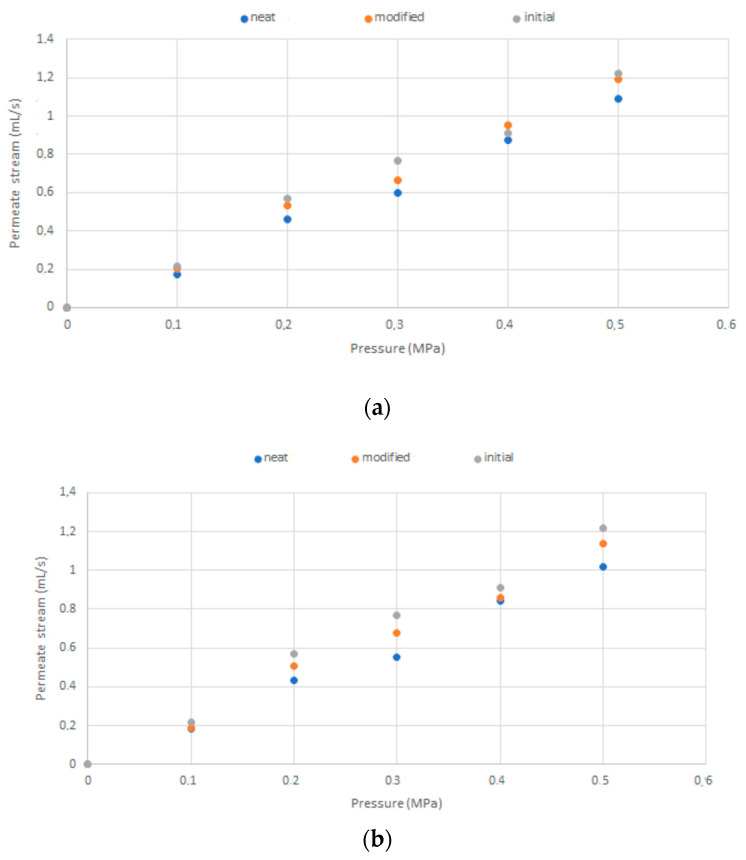
The water permeate stream flow rate as a function of pressure at 20 °C—mean value based on three measurements, maximal average RD = 7.5%. (**a**) Neat and modified membrane after incubation in *S. aureus* broth culture; (**b**) neat and modified membrane after incubation in *E. coli* broth culture.

The membrane’s blockage in contact with *S. aureus* and *E. coli* was evaluated in the same cross-filtration system set up. The neat membranes and membranes with 20% of thymol were incubated for a week in the presence of *E. coli* and *S. aureus* growing cultures. After the incubation, the water permeate flow rates through both types of incubated membranes and the control (initial commercial membrane) were determined as a function of the pressure. The results are presented in Figure 8. In the case of incubation with *S. aureus* (Figure 8a)*,* the impregnated membrane behaved similarly to the control membrane (initial). At the same time, in the neat bacteria-exposed membrane, a decrease in the permeate stream flow rate was detected, indicating its blockage due to adhesion of *S. aureus*. After the incubation of membranes with *E. coli,* the reduction of the permeate flow rate was observed in both membranes (Figure 8b). However, the decline was considerably lower for the thymol-impregnated membrane compared with the neat membrane. These results suggest that the impregnated membranes were less prone to blockage with bacteria than neat ones.

The quality status of membranes before and after the incubation in the presence of *E. coli* and *S. aureus* growing cultures was expressed by their resistance (*R_tot_ = R_m_ + R_f_*) to water filtration calculated from the modified form of Darcy’ equation (5) [38,39]. The results are presented in Table 2. As can be seen, the neat and modified (20% thymol) membranes without incubation had similar resistances to water filtration (7.12 and 7.05, respectively), proving that the SSI had not affected the membrane’s structure. After the static exposure to bacterial growing cultures, the resistance increased for the *R_f_* value (Table 2). The *R_f_* values’ comparison reveals that the resistance increase was considerably smaller for the modified membranes than the neat ones (54% smaller for *E. coli*, and 70% smaller for *S. aureus*) because of the added antibacterial properties.

## 3. Materials and Methods

**Figure 9:**
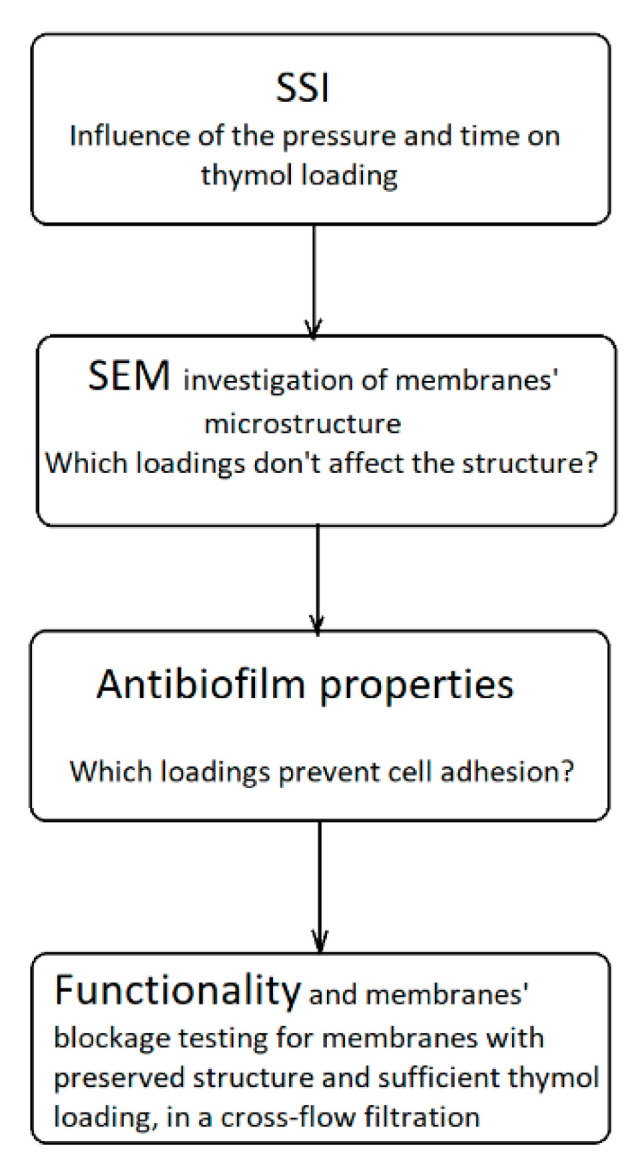
Schematic presentation of the research organization.

The research comprised four stages (Figure 9). In the first stage, the potential of the SSI to modify CA membranes was explored. Thymol loadings were determined as a function of pressure and time in a batch impregnation process. In the next step, scanning electron and ion microscopy was applied to investigate the SSI’s influence on membranes’ microstructure. The task was to identify high thymol loadings that do not affect the structure. The third stage aimed to investigate the antibiofilm properties of impregnated membranes against selected strains. Besides standardized microbiological procedures, scanning electron microscopy was employed to observe the biofilm presence at the membranes’ surface. The task was to identify thymol loadings that provide strong antibiofilm properties. Based on the second and third stage results, membranes with retained structures and sufficient thymol quantities to provide strong antibacterial properties were selected for the functionality testing in a cross-filtration system and comparison with the untreated CA membranes (stage four). The filtration process was modeled to quantify membranes’ performance. In this stage, the membranes’ blockage in static contact with selected strains was also investigated.

### 3.1. Materials

Commercial cellulose acetate (CA) microfiltration membranes with 0.2 µm average pore diameter and 47 mm membrane diameter were supplied by GE Healthcare Whatman TM, Hino & Toky, Japan (Cat. No. 7001–0004). Thymol (purity ≥ 98.5%) was purchased from Sigma-Aldrich, Darmstadt, Germany. Carbon dioxide (purity > 99.99%) was provided by Air Liquid, Wroclaw, Poland.

### 3.2. Supercritical Solvent Impregnation (SSI)

Commercial CA membranes were impregnated with thymol in a 280 mL volume high-pressure vessel (Eurotechnica GmbH, Bargteheide, Germany) equipped with a heating jacket, where water was used as the heating fluid. A heating bath circulator (Jeio Tech Co., Ltd., Daejeon, Korea) was employed to recirculate water and maintain its temperature. The SSI was performed at a temperature of 40 °C and pressures of 10, 15, and 20 MPa. The processing time was varied from 0.5 h to 6 h. Around 1.8 g of thymol was put in a glass container and placed in the middle of the vessel. Two membranes were put to the left of the thymol, and two were put to the right. Upon reaching the desired temperature, carbon dioxide was pumped into the system by an air-driven gas booster (Eurotechnica GmbH, Bargteheide, Germany), and the system was left under the operating conditions for the impregnation. A moderate decompression rate of 0.25 MPa/min followed. All experiments were performed in triplicates. The impregnation yield (thymol loading) was determined gravimetrically and calculated according to the equation:

(1) $$Y\;=\;\frac{m_{th}}{m_{im}}\;\times \;100\%$$

where *m_th_* is the mass of impregnated thymol obtained as a mass difference between the impregnated and neat membrane, and *m_im_* is the mass of the impregnated membrane. Membranes were also exposed to scCO_2_ without impregnation, and the quantity of extractable matter was found to be negligible.

All the experiments were performed in five replicates, and the standard deviation was calculated according to the formula

(2) $$SD\;=\;\sqrt{\frac{\sum {\left(Y_{i}\;-\;Y_{av}\right)}^{2}}{N}}$$

where *Y_i_* is the variable value (impregnation yield) obtained in experiment *i*, *Y_av_* is the average value of the variable, and *N* is the number of experiments.

### 3.3. FTIR Analyses

The Fourier-transform infrared (FTIR) spectroscopy analysis was performed to confirm the presence of thymol on the impregnated membrane’s surface. The spectra of the neat and thymol impregnated membranes were recorded in ATR mode using Nicolet iS50 Spectrometer (Thermo Fisher SCIENTIFIC, Waltham, MA, USA) with a resolution of 4 cm^−1^ at wavenumbers in the range of 500–4000 cm^−1^.

### 3.4. Scanning Electron Microscopy (SEM)

The structural properties of neat and impregnated membranes were investigated using a two-beam microscope SEM/Ga-FIB FEI Helios NanoLab™ 600i (FEI, Thermo Fisher Scientific, Eindhoven, The Netherlands, which comprises ultra-high resolution electron and ion microscopy. Energy focused beam of gallium ions allows for the selective removal of the preparation material and modification at the nanoscale, which provides the ability to perform the sample cross-sections. Before the analyses, the samples were coated with gold.

### 3.5. Antibacterial Analyses

#### 3.5.1. Bacterial Strains

The strains used in this study were *Staphylococcus aureus* ATCC 25923, *Staphylococcus aureus* ATCC 43300 (methicillin-resistant *S. aureus*), and *Pseudomonas aeruginosa* PAO1 NCTC 10332. The bacteria were grown in brain heart infusion (BHI) medium at 37 °C. Biofilms of *S. aureus* were grown in BHI supplemented with 0.5% glucose.

#### 3.5.2. Analysis of Anti-Biofilm Properties of Impregnated Membranes

The effect of thymol impregnation on bacterial attachment and biofilm formation at the surface of CA membranes surfaces was evaluated using static biofilm formation assay in 24-well microtiter plates (Sarstedt, Nümbrecht, Germany). Overnight cultures of bacteria were diluted to 5 × 10^7^ cells per mL in an appropriate medium, and 1 mL was added to each well containing a square piece (1 cm^2^) of either thymol impregnated or non-impregnated membranes, which served as the negative control. After incubation for 24 h at 37 °C, the membranes were removed from the wells. The quantification of biofilms formed on the membranes’ surfaces was as follows: each membrane piece was extensively washed with sterile phosphate-buffered saline pH 7.2–7.4 (PBS), and then adhering cells were detached into PBS by sonication using an ultrasonic water bath (SONIC, Ultrasonic cleaner, SONIC, Niš, Serbia) at the frequency of 40 kHz. The films were then immersed into 600 µL PBS and sonicated for 5 min, followed by 5 min vortexing at 500 rpm at room temperature. This procedure was repeated two times, and the collected cells (in 1.2 mL of PBS) were thoroughly vortexed and ten-fold serially diluted. Appropriate dilutions were plated on BHI agar plates in triplicates, and after 24 h of incubation at 37 °C, formed colonies were counted. Sterility of the membranes was checked by incubation of the membranes in 1 mL BHI for 24 h at 37 °C and monitoring of bacterial growth in the medium by measuring optical density (OD_600nm_) and at the membrane surface as described above. The experiment controls also included bacterial growth control (inoculated medium without membranes) and the medium sterility control. The experiments were performed in triplicates, and the measurements were repeated two times.

#### 3.5.3. Scanning Electron Microscopy (SEM) for Biofilm Detection

A field emission scanning electron microscopy (FESEM, Tescan Mira3 FEG, Tesacan, Brno, Czech Republic) was used to visualize *S. aureus* ATCC 25923 adhesion on the membranes’ surfaces. The overnight bacterial cultures were diluted to 5 × 10^7^ cells per mL in BHI medium supplemented with 0.5% glucose, and 1 mL was added per well in 24-well microtiter plates. The membranes of 1 cm^2^ in surface area, neat and impregnated, were placed in wells containing bacteria and incubated for 24 h at 37 °C. Then, the culture medium was removed, and the membranes were washed three times in PBS to remove the non-adherent bacteria. After rinsing with water, the adherent cells were fixed with cold methanol for 20 min and the samples dried before the examination. The samples were coated with gold before the analyses.

#### 3.5.4. Statistical Analysis

Excel statistical software 2016 MSO (Student’s *t*-test) was used to determine the statistical significance of anti-biofilm properties of impregnated membranes. The level of statistical significance was defined as *p* < 0.05 and is presented in Table 1 with an asterisk (**).

### 3.6. Membranes’ Functionality Testing

The membranes’ functionality was tested in cross-flow filtration experiments with *S. aureus* DSM 2569 and *E. coli* DSM 4509. The bacteria were cultured in 500 mL flasks in 160 mL of appropriate medium. Each medium was inoculated with 10 mL of 48 h old culture and incubated at 37 °C at 180 rpm (IKA KS 4000). The medium for *S. aureus* contained the following (in grams per liter): casein hydrolysate 10, thiamine 0.26, nicotinamide 0.05, Na_2_HPO_4_ × 12 H_2_O 15.2, KH_2_PO_4_ 3.0, NaCl 0.5, NH_4_Cl 1.0, MgSO_4_ × 7H_2_O 0.25, and CaCl_2_ 0.01. The medium for *E.coli* included the following (in grams per liter): glucose 6.0, Na_2_HPO_4_ × 12 H_2_O 15.2, KH_2_PO_4_ 3.0, NaCl 0.5, NH_4_Cl 1.0, MgSO_4_ × 7H_2_O 0.25, and CaCl_2_ 0.01. The tests were performed with OD_600_ in the range from 0.12 to 0.58.

**Figure 10:**
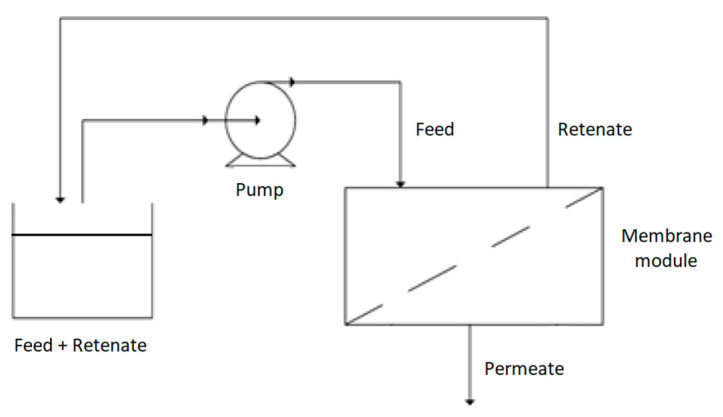
The scheme of the cross-flow filtration system.

Evaluation of whether there is an adverse effect in the modified membrane’s functionality due to the polymer swelling was carried out with *E. coli*. The filtration of *E. coli* culture broth was performed in a laboratory cross-flow filtration system presented in Figure 10 with two different cell concentrations (25.2 × 10^6^ and 115.9 × 10^6^ CFU/mL). The permeate stream flow rates through the neat membrane and membrane with 20% of thymol were measured.

To evaluate membranes’ blockage due to exposure to bacterial cells, modified (20% of thymol) and neat membranes were incubated under the static condition for one week at 37 °C in the presence of *S. aureus* (volume 150 mL, initial cell concentration 14.5 × 10^6^ CFU/mL) and *E. coli* culture (volume 150 mL initial cell concentration 14.8 × 10^6^ CFU/mL). After the incubation period, the membranes were rinsed twice with distilled water, and the permeate flow rates of water through the incubated membranes and the untreated control membrane were recorded for different pressures. The experiments were completed using the experimental set-up presented in Figure 10.

All the experiments in the cross-flow filtration system were performed in triplicates. The standard deviation (SD) was calculated according to Equation (2), while the average relative deviation was calculated as the average of relative deviations (RD, Equation (3)) for three experiments.

(3) $$RD\;=\;\frac{\left|Y_{i}\;\;-\;\;Y_{av}\right|}{Y_{av}}\;\times \;100\%$$

#### 3.6.1. Mathematical Modeling of the Cross-Flow Filtration

The permeate flux decline in microfiltration due to fouling can be expressed by the following equation [36,37]:

(4) $$\;\;-\;\;\frac{dJ}{dt}=\alpha \;\times \;J^{3\;\;-\;\;\beta }$$

where *J* is the permeate flux (m^3^/(m^2^ s)); *t* is the time (s); *α* is the kinetic constant (1/s); (3 − *β*) is the reaction order; and *β* is the constant with a value 0, 1, 3/2, or 2. Each value of the parameter *β* corresponds to a particular membrane blocking mechanism [36,37].

The presented model was used to simulate the permeate flux decline in the cross-filtration of *E. coli* culture broth. The value of the kinetic constant *α* was estimated using the non-linear least-squares method separately for each possible fixed value of *β*. The numerical solution of the model differential Equation (4) was performed using the Runge–Kutta method and fitted to the experimental data. The error function minimum was found using the Nelder–Mead simplex direct search method in MATLAB. The goodness of fits was characterized by the coefficient of determination (R^2^).

#### 3.6.2. The Incubated Membranes’ Resistance Determination

The effects of the cell adhesion to the membranes’ surface during their static exposure to *S. aureus* and *E. coli* growing cultures were quantified by a modified form of Darcy’s Equation (5), used to determine the membranes’ resistance in water filtration tests [38,39].

(5) $$J=\frac{\Delta P}{\mu \cdot R_{tot}}=\frac{\Delta P}{\mu \cdot (R_{m}+R_{f})}$$

where *J* is the permeate flux (m^3^/(m^2^ s)), Δ*P* is the transmembrane pressure (Pa), *µ* is the dynamic viscosity (Pa s), *R_tot_* is the total membrane resistance (with adhered contaminants) (m^−1^), *R_m_* is the membrane resistance (m^−1^), and *R_f_* is the cake (adhered material) resistance (m^−1^).

## 4. Conclusions

The study has proved that commercial cellulose acetate microfiltration membranes can be efficiently impregnated with high thymol quantities by SSI without disrupting their structure and functionality. It has been shown that thymol loading of 20% completely halted the formation of *S. aureus* biofilms and reduced by ten-fold *P. aeruginosa* adhesion to CA membrane. Consequently, modified membranes were less prone to blockage when exposed to bacteria. It was demonstrated that such modification of the commercial microfiltration membranes’ defined polymeric structure could be performed in a short (30 min) and environmentally friendly process. The membranes’ properties depended on thymol loading and not on the processing pressure applied to load the material. The modified membranes with strong antibacterial activity might have applications in the biomedical field, venting and protecting medical devices and equipment, and intensive care and surgery rooms’ ventilation.

## Figures and Tables

**Table 1:** Inhibition of bacterial adhesion at the surface of neat and thymol impregnated cellulose acetate (CA) membranes. Values are presented as mean ± SD.

Membrane	*S. aureus* ATCC 25923	*S. aureus* MRSA ATCC 43300	*P. aeruginosa* PAO1
	CFU */cm^2^ (×10^6^)		
CA neat	1.2 ± 0.3	16.2 ± 2	26.7 ± 9.3
CA + 20% thymol	0	0	2.4 ± 0.2 **
CA + 30% thymol	0	0	0.0022 ± 0.0001 **

* CFU—colony forming unit; MRSA—methicillin-resistant *S. aureus*. ** Statistically significant difference of anti-biofilm properties between control (neat) and corresponding thymol-impregnated membrane (*p* < 0.05).

**Table 2:** Resistance to water filtration of the membranes before and after incubation in the presence of *E. coli* and *S. aureus* growing cultures, given as mean ± SD.

Membrane	*R_tot_* × 10^−11^ (m^−1^)	*R_f_* × 10^−11^ (m^−1^)
Initial neat CA *	7.12 ± 0.54	-
Modified CA *	7.05 ± 0.49	-
Neat (*E. coli*)	8.53 ± 0.31	1.41 ± 0.031
Modified (*E. coli*)	7.70 ± 0.32	0.654 ± 0.032
Neat (*S. aureus*)	8.06 ± 0.47	0.938 ± 0.047
Modified (*S. aureus*)	7.33 ± 0.29	0.281 ± 0.029

* Without incubation.

## Data Availability

The data presented in this study are available on request from thecorresponding author.

## Sample Availability

Samples are available from the corresponding author.

## Appendix A

**Table A1:** Impregnation yields for SSI of commercial CA membranes at 40 °C.

t (h)	10 MPa	15 MPa	20 MPa
0.5	12.2 ± 1.1	21.2 ± 0.8	22.6 ± 1.2
1	21.2 ± 0.9	23.4 ± 1.3	30.9 ± 1.9
2	27.0 ± 1.2	30.1 ± 1.9	35.5 ± 1.4
3	31.9 ± 2.1	36.0 ± 1.5	42.0 ± 0.3
4	36.7 ± 1.8	42.8 ± 0.4	
6	41.3 ± 0.9		
